# Supplementary figures and images for: Joint modeling of effect sizes for two correlated traits: Characterizing trait properties to enhance polygenic risk prediction
Source: PLoS Genet. 2026 Jan 26;22(1):e1012026. doi: 10.1371/journal.pgen.1012026 (PMC12858075; doi:10.1371/journal.pgen.1012026)

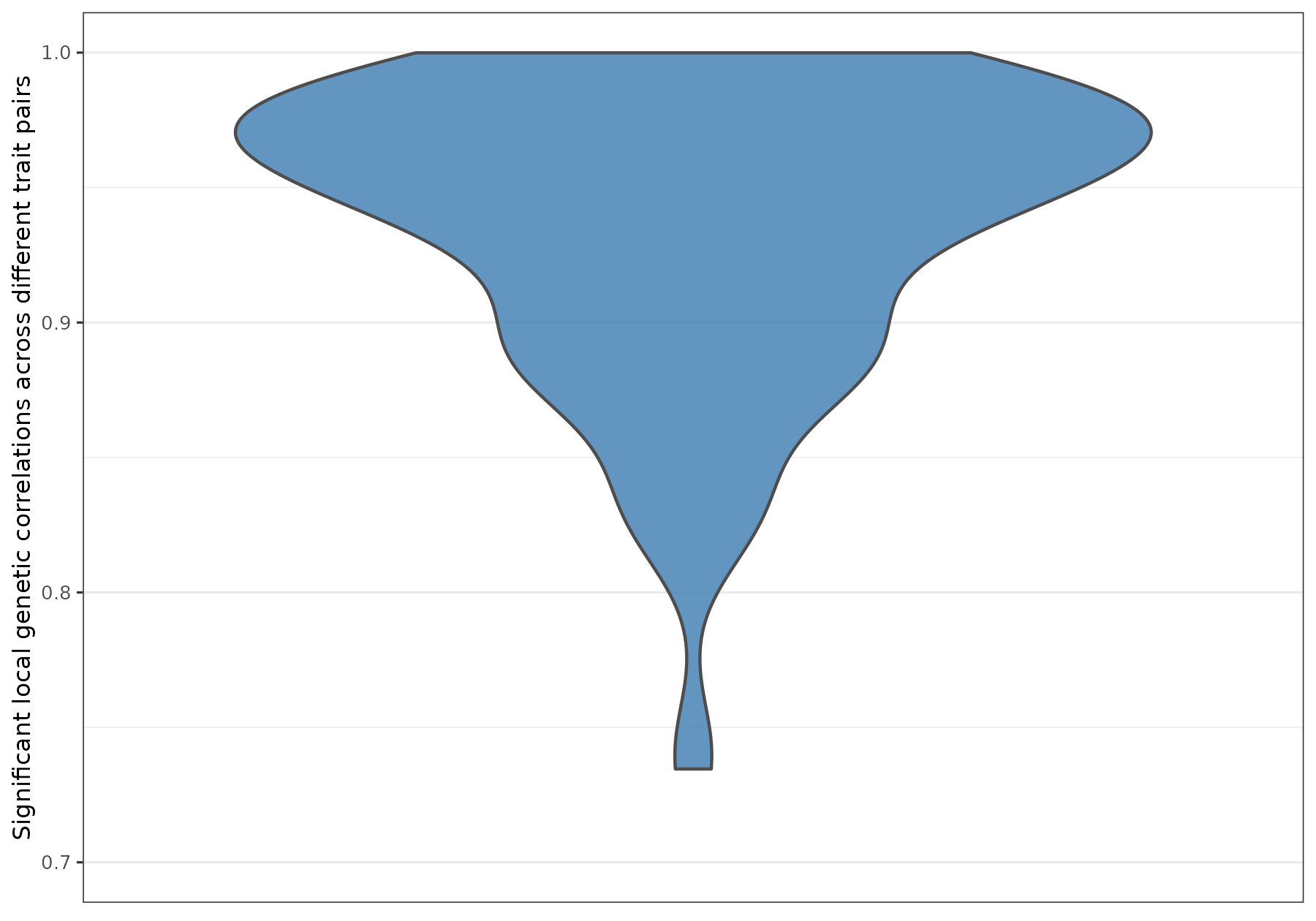

Supplement: S1 Fig — Local genetic correlations are estimated by SUPERGNOVA and the significant local genetic correlations are selected with pvalue < 0.05/number of regions used for each trait pair. The above distribution of local genetic correlations integrates results from all trait-pairs used in the real application. (TIF) [file pgen.1012026.s002.tif]

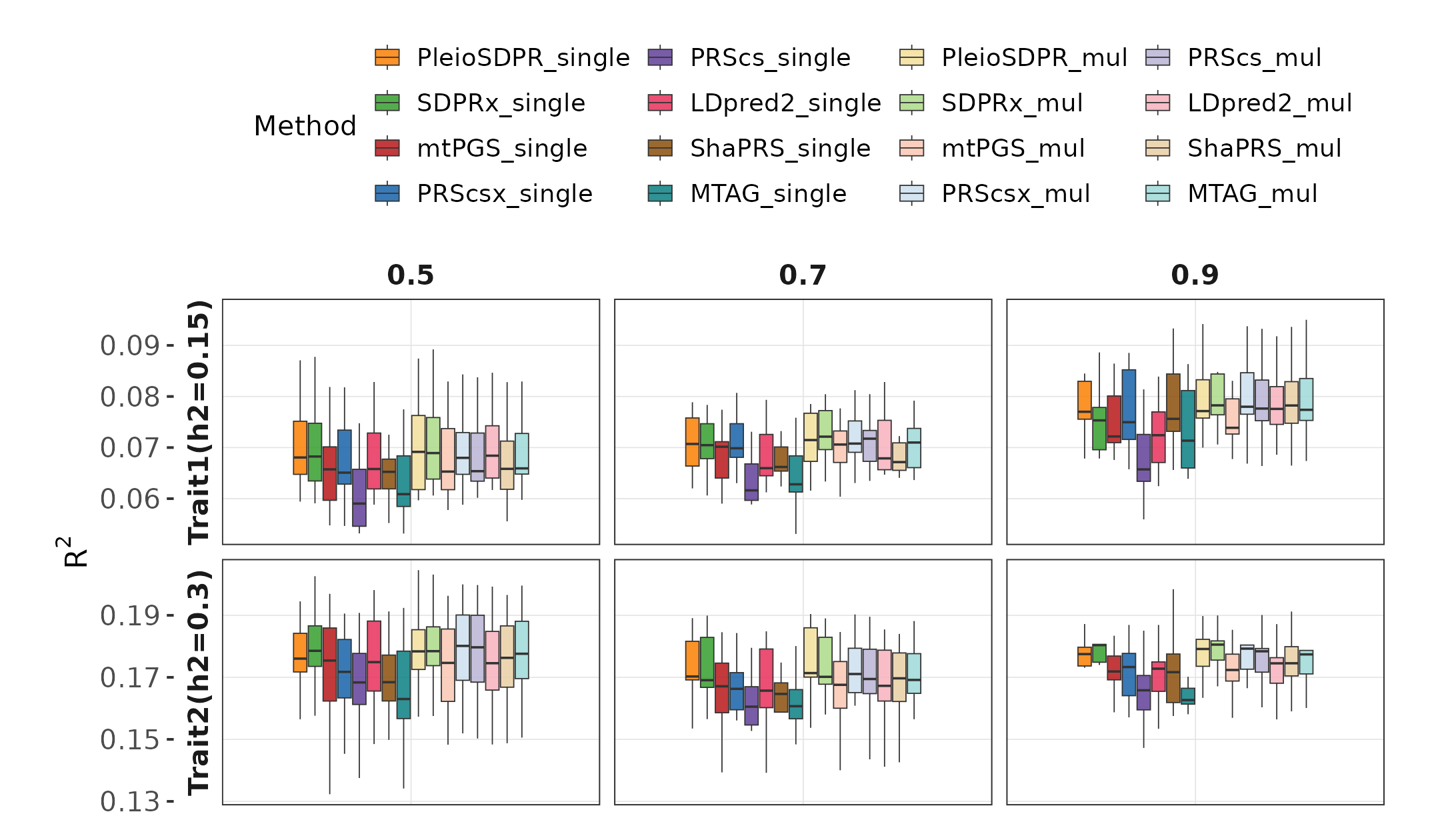

Supplement: S2 Fig — We compare the methods considering with validation dataset and without validation dataset. When there is no validation dataset, we name them as PleioSDPR_single, SDPRx_single, mtPGS_single, PRScsx_single, PRScs_single, LDpred2_single, ShaPRS_single and MTAG_single. When there is a validation dataset, we name them PleioSDPR_mul, SDPRx_mul, mtPGS_mul, PRScsx_mul, PRScs_mul, LDpred2_mul, ShaPRS_mul and MTAG_mul. Exact R2values for all methods are provided in S5 Table. (TIF) [file pgen.1012026.s003.tif]

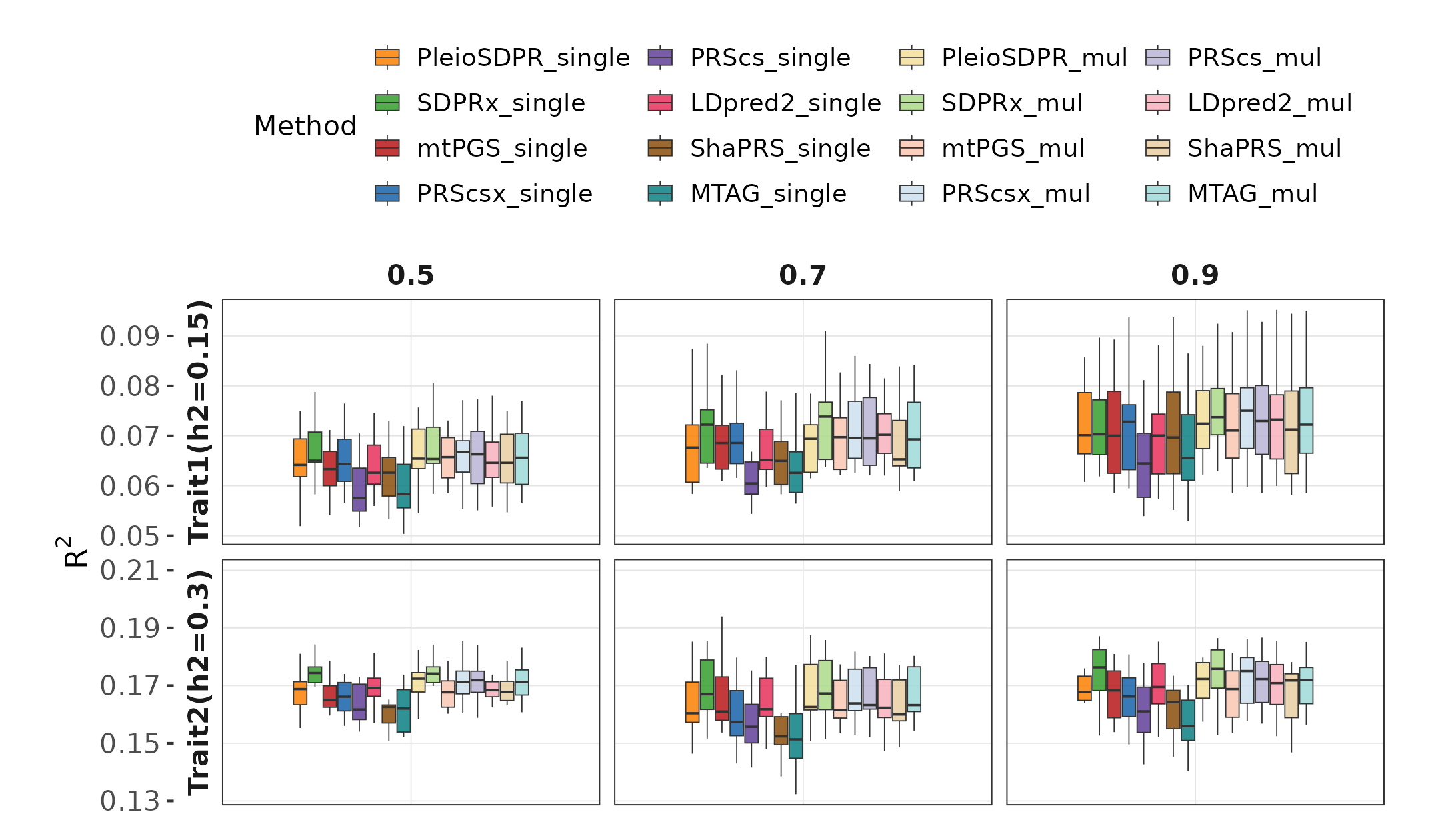

Supplement: S3 Fig — We compare the methods considering with validation dataset and without validation dataset. When there is no validation dataset, we name them as PleioSDPR_single, SDPRx_single, mtPGS_single, PRScsx_single, PRScs_single, LDpred2_single, ShaPRS_single and MTAG_single. When there is a validation dataset, we name them PleioSDPR_mul, SDPRx_mul, mtPGS_mul, PRScsx_mul, PRScs_mul, LDpred2_mul, ShaPRS_mul and MTAG_mul. Exact R2 values for all methods are provided in S6 Table. (TIF) [file pgen.1012026.s004.tif]

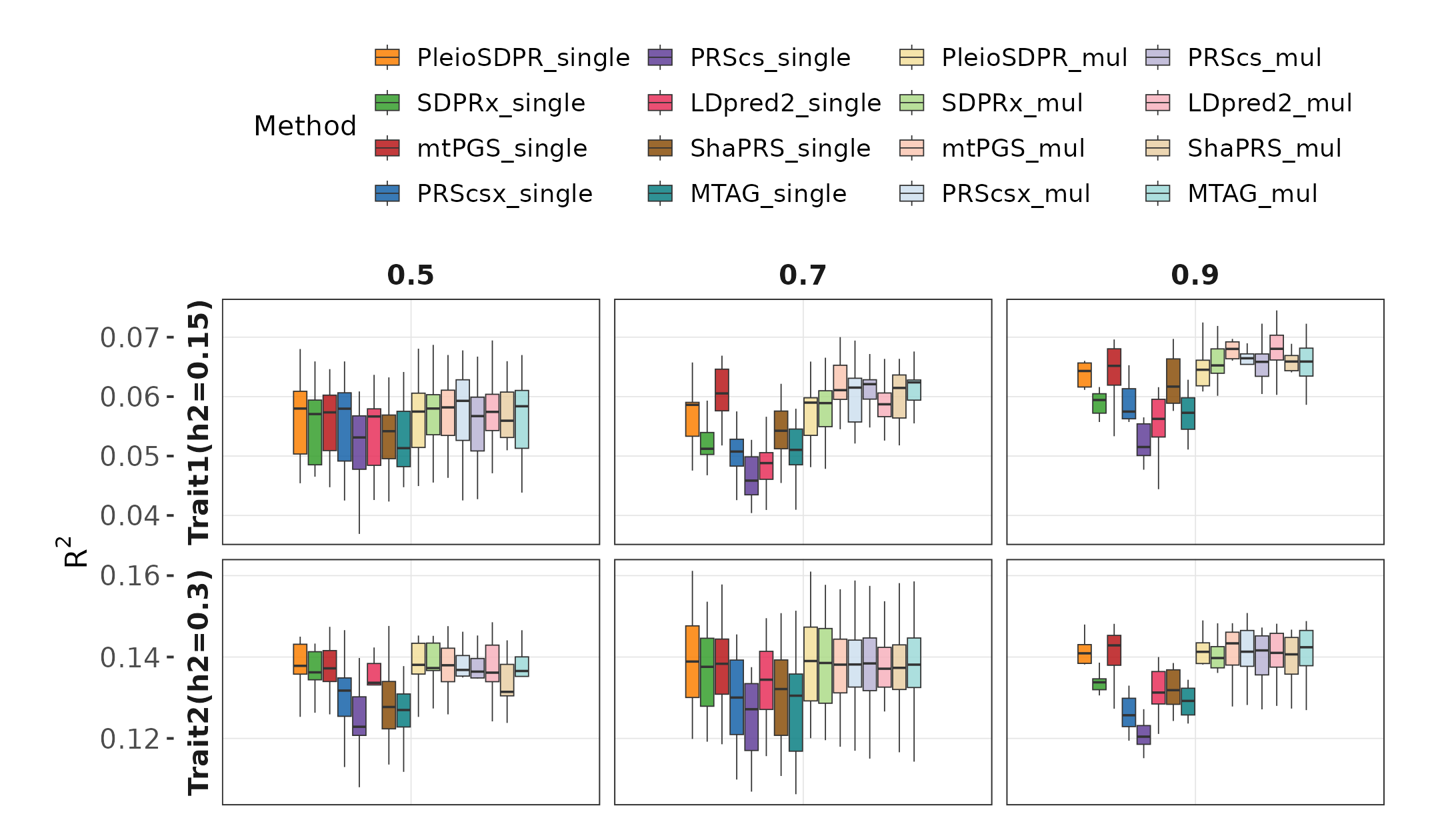

Supplement: S4 Fig — We compare the methods considering with validation dataset and without validation dataset. When there is no validation dataset, we name them as PleioSDPR_single, SDPRx_single, mtPGS_single, PRScsx_single, PRScs_single, LDpred2_single, ShaPRS_single and MTAG_single. When there is a validation dataset, we name them PleioSDPR_mul, SDPRx_mul, mtPGS_mul, PRScsx_mul, PRScs_mul, LDpred2_mul, ShaPRS_mul and MTAG_mul. Exact R2values for all methods are provided in S7 Table. (TIF) [file pgen.1012026.s005.tif]

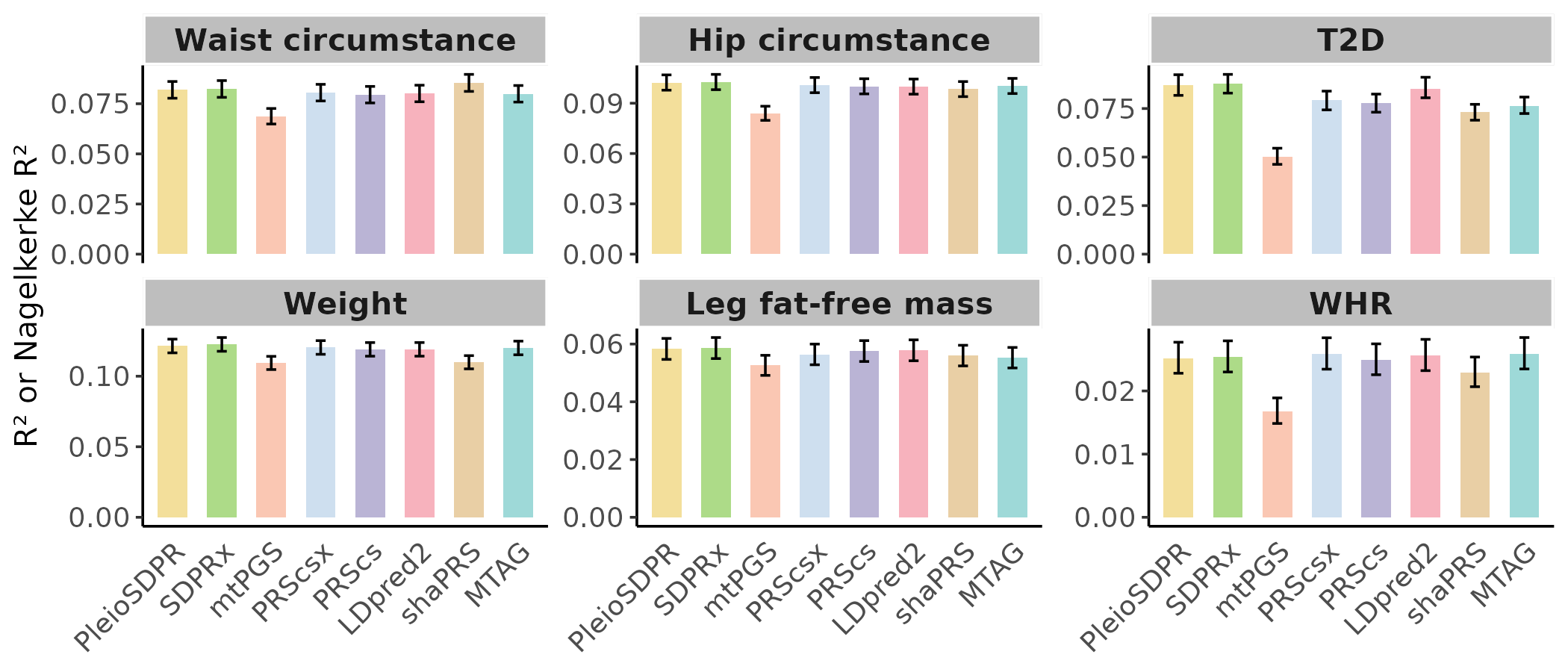

Supplement: S5 Fig — Error bars represent 95% confidence intervals. (TIF) [file pgen.1012026.s006.tif]

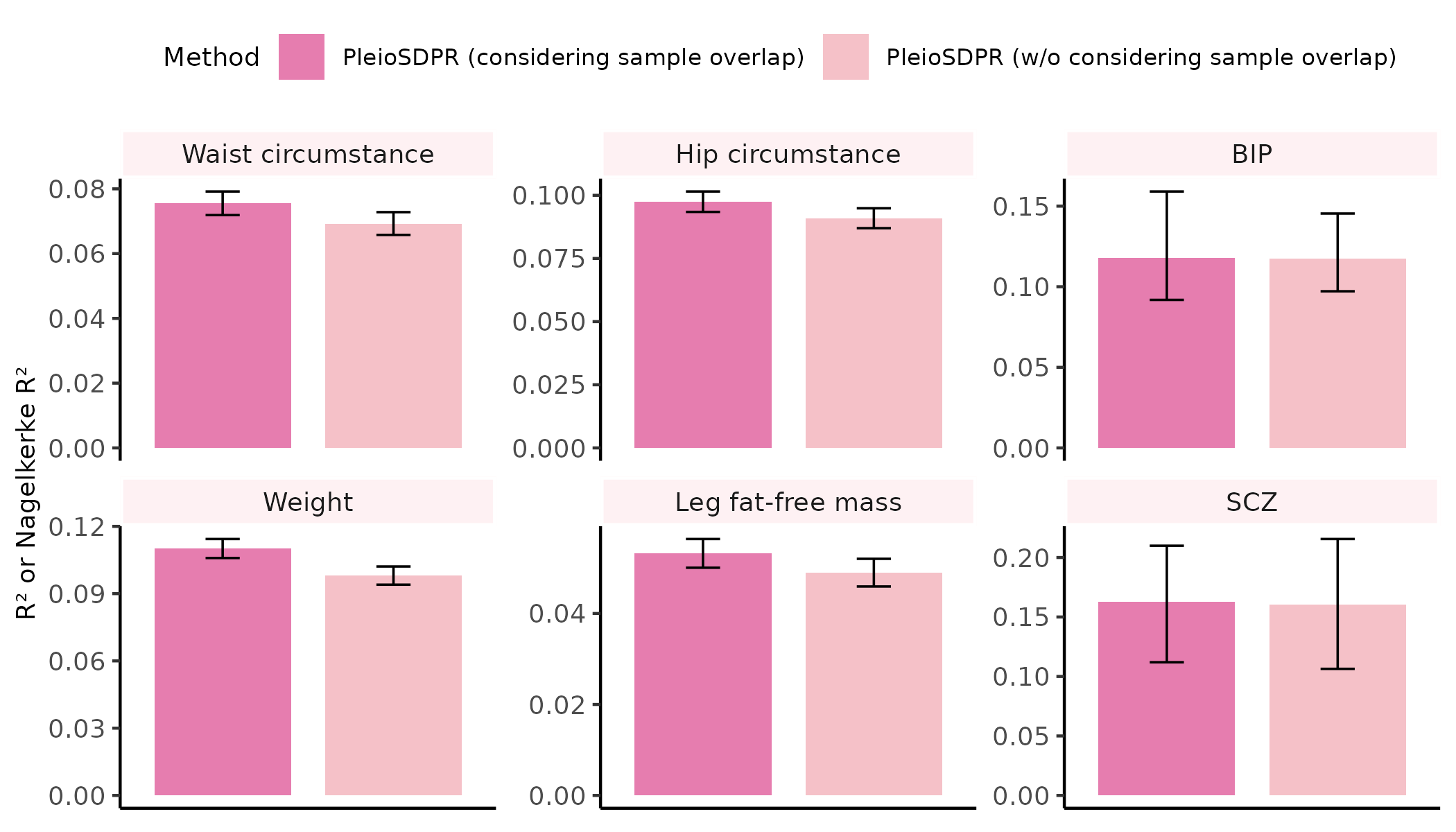

Supplement: S6 Fig — Error bars represent 95% confidence intervals. (TIF) [file pgen.1012026.s007.tif]

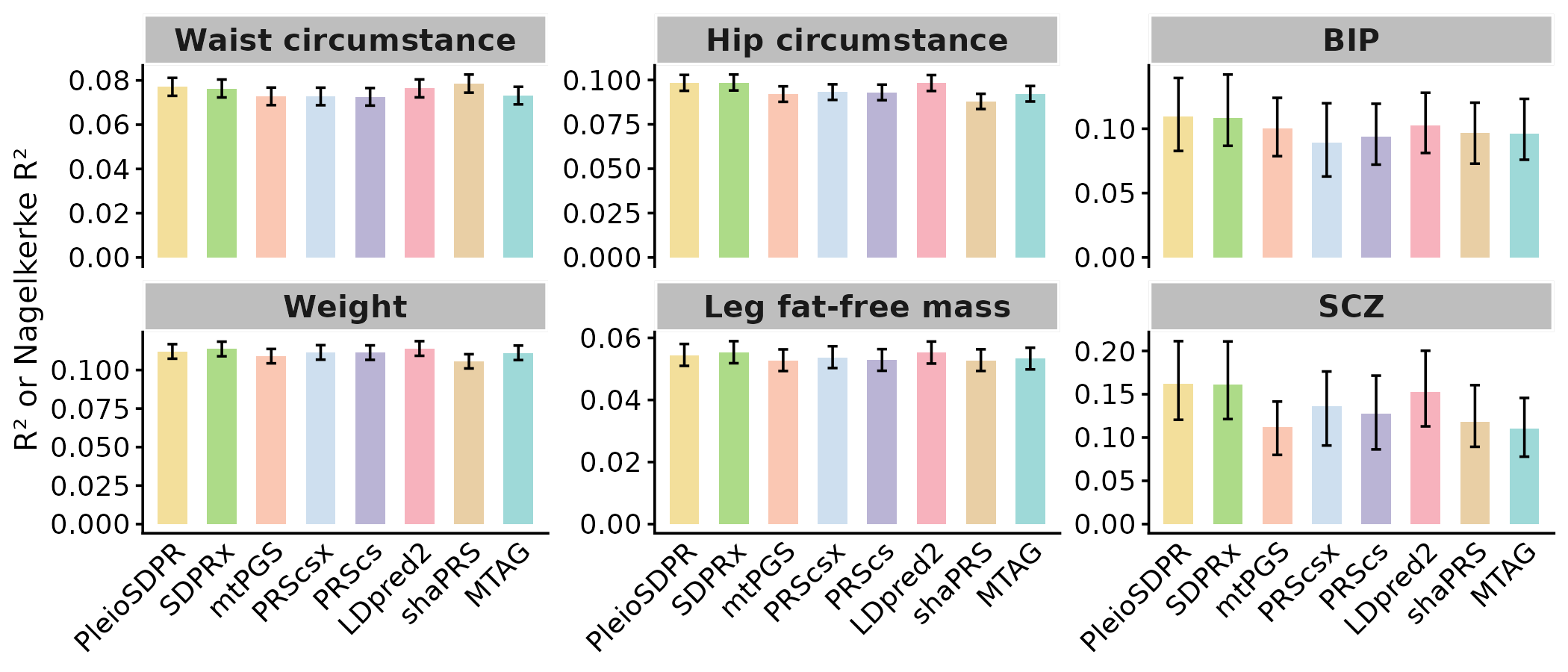

Supplement: S7 Fig — Error bars represent 95% confidence intervals. (TIF) [file pgen.1012026.s008.tif]

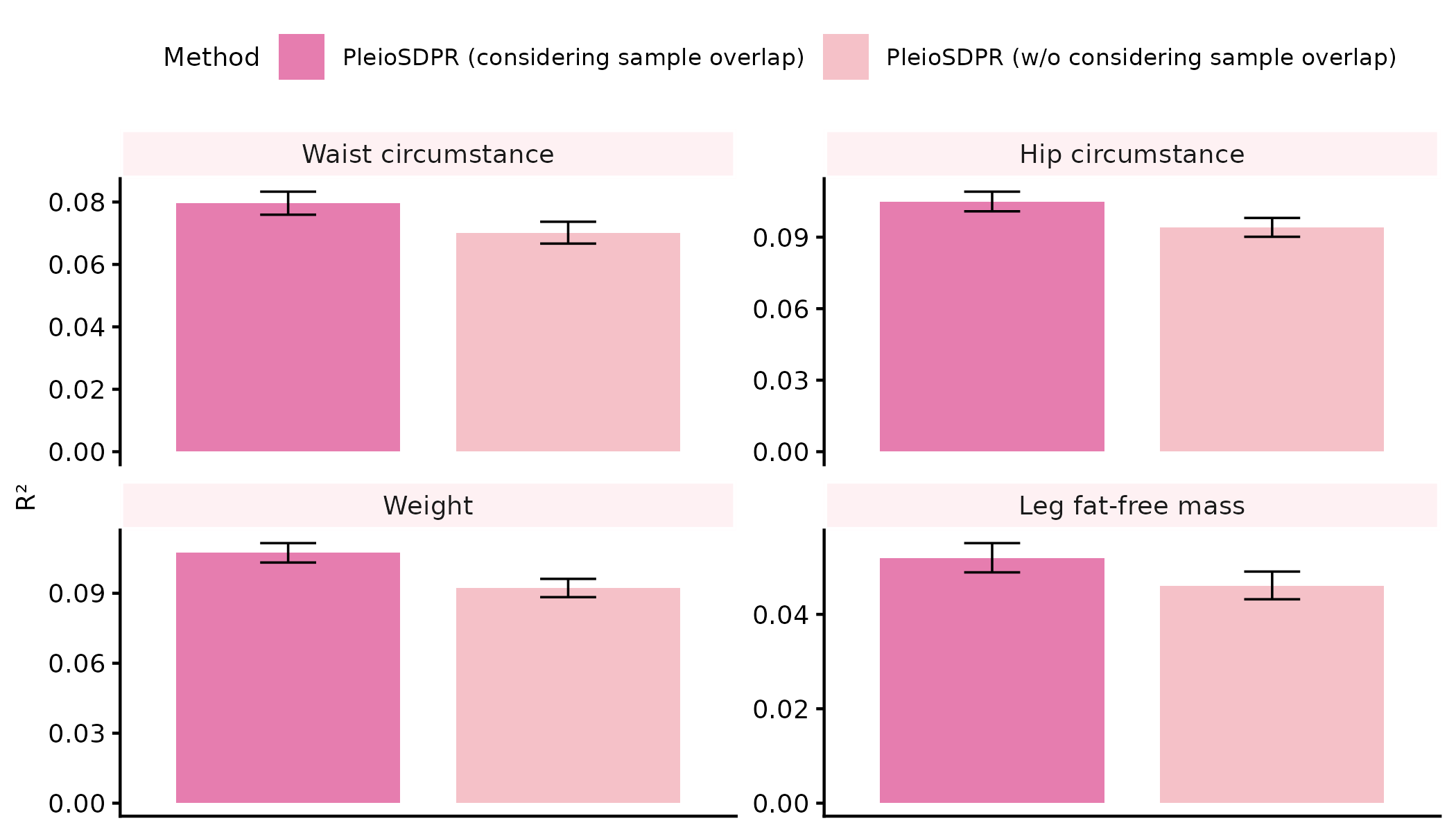

Supplement: S8 Fig — Error bars represent 95% confidence intervals. (TIF) [file pgen.1012026.s009.tif]

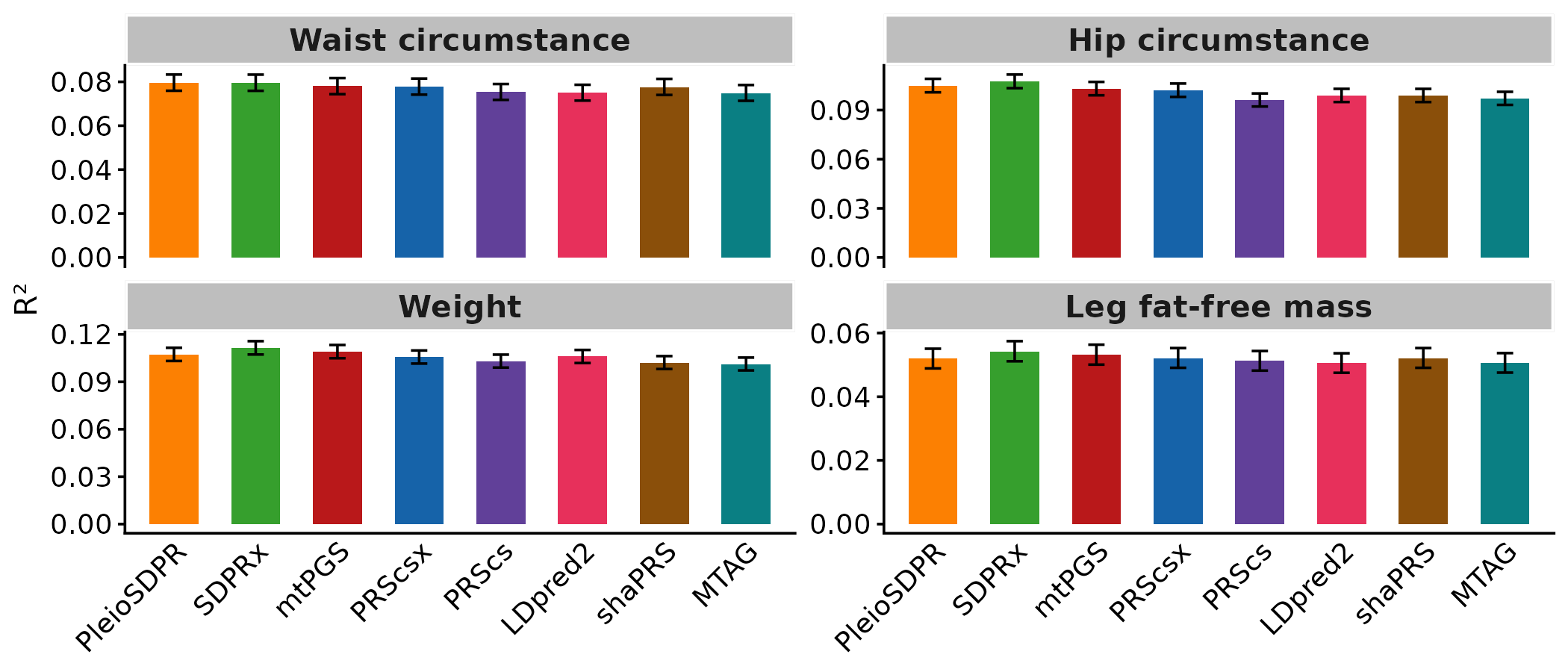

Supplement: S9 Fig — Error bars represent 95% confidence intervals. (TIF) [file pgen.1012026.s010.tif]

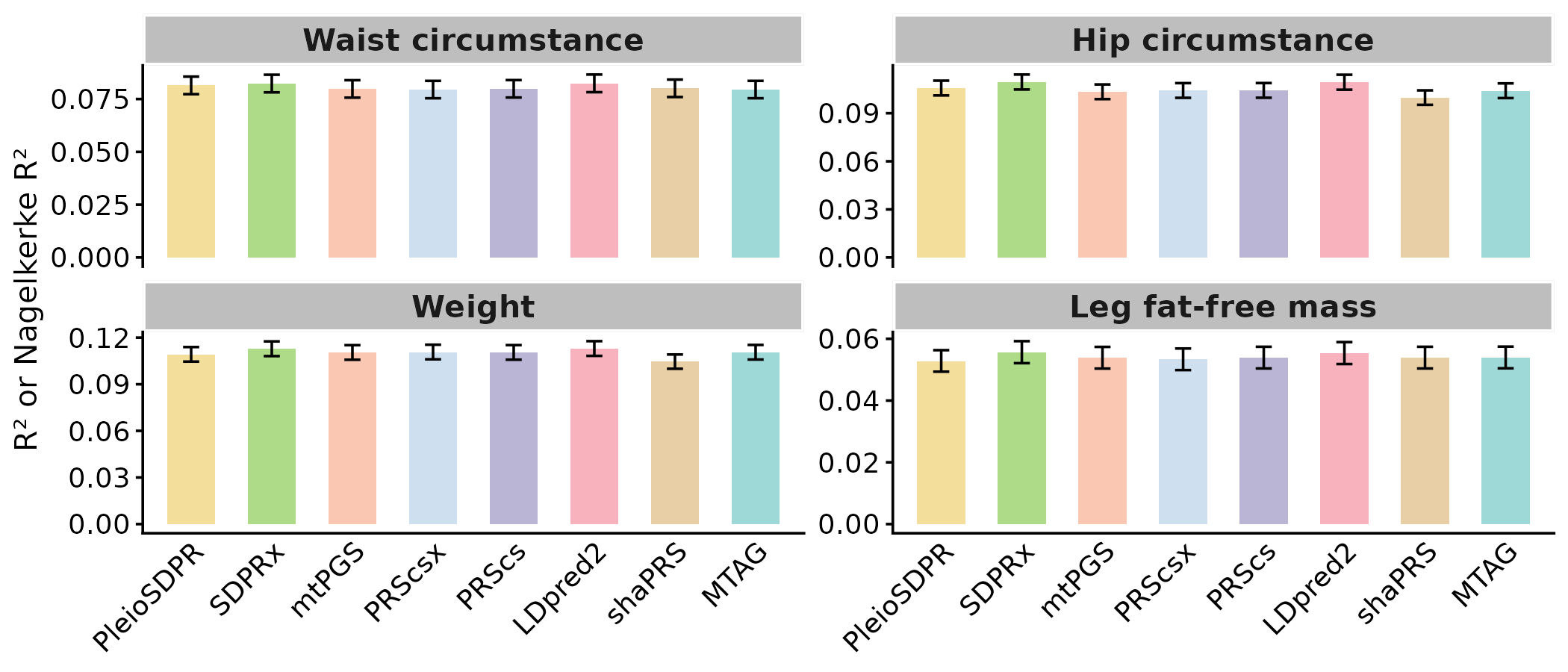

Supplement: S10 Fig — Error bars represent 95% confidence intervals. (TIF) [file pgen.1012026.s011.tif]
