# Supplementary material for: Joint modeling of effect sizes for two correlated traits: Characterizing trait properties to enhance polygenic risk prediction
Source: PLoS Genet. 2026 Jan 26;22(1):e1012026. doi: 10.1371/journal.pgen.1012026 (PMC12858075; doi:10.1371/journal.pgen.1012026)
Supplement: Attachment — Submitted filename: Response to reviewers.docx [file pgen.1012026.s021.docx]

**Respond to Reviewer #1**

**We sincerely thank you for the constructive and insightful comments. Below, we provide point-by-point responses and describe the corresponding revisions made in the manuscript.**

**Major Comments**

1. Page 11, line 145: In the part of “Compared Methods”, the authors applied PRS-CSx and SDPRX to represent multivariate PGS method. However, both of them are designed for cross-ancestries PGS model but not cross-traits. The famous and representative cross-traits PGS category did not be considered, like MTAG (2017, NG) or shaPRS (2024, AJHG), which firstly integrating GWAS summary statistics of two traits and then apply single-trait PGS method. Could the authors add these methods for fair comparison?

**Answer:** Thank you very much for pointing this out. We agree with the reviewer that PRScsx and SDPRx were originally developed for cross-ancestry polygenic prediction. However, after modifying the reference panels to be from the same population and providing the GWAS summary statistics of two traits as input, both methods can also be applied to a cross-trait setting. Conceptually, because these methods are designed to borrow information across correlated effect-size distributions, they can also incorporate information from correlated traits under a cross-trait setting. To clarify this rationale, we have added the following sentences to the manuscript in the introduction part and highlighted them with a yellow background in the document ‘Revised Manuscript with Track Changes’:

*“Although many multivariate PGS models were initially developed for cross-population prediction, their underlying modeling frameworks are not restricted to population differences. These methods jointly model multiple GWAS inputs by assuming partially shared effect-size distributions and by applying coupled shrinkage across the input datasets. When the reference panels are applied to the same population, the cross-study information sharing in these models effectively becomes cross-trait sharing. In other words, when the two input GWAS correspond to different traits rather than different populations, the Bayesian effect-size sharing mechanism still allows each trait to borrow statistical strength from the other. This enables PRScsx and SDPRx to function as multivariate PGS approaches in a cross-trait context, leveraging pleiotropy to improve prediction performance.”*

Thank you also for highlighting MTAG and shaPRS. We have now included both methods in our comparison. We first used MTAG and shaPRS to integrate GWAS summary statistics on two traits and then used PRScs to calculate the PGSs. The results from MTAG and shaPRS have been added to both the simulation studies and the real-data analysis.

2. Page12, line 167. In the part of “Simulations”, the authors fixed the GWAS sample size to be 20,000 among all simulations without exploring other sample size scenarios. The GWAS sample size may affects the robustness of local (and global) genetic correlation between two traits, which may enhance the performance of your method. Could the authors add the corresponding scenarios or explain why alternative GWAS sample size scenarios were not explored?

**Answer:** Thank you for this valuable suggestion. We agree that GWAS sample size can influence the estimation of local and global genetic correlation, which in turn may affect the performance of multivariate PGS methods. In response to the reviewer’s comment, we have now conducted an additional simulation scenario in which both GWASs have a sample size of 10,000 individuals per trait, with no sample overlap. These results are now included in Supplementary Figure S4, and a brief description has been added to the Simulation section to clarify this extension, highlighted in yellow in the document ‘Revised Manuscript with Track Changes’.

*“To assess the impact of sample size on model performance, we additionally generated sets 4 and 5, each consisting of 10,000 non-overlapping individuals.”*

*“To evaluate the influence of GWAS sample size on model performance, we conducted an additional simulation in which each trait had a sample size of 10,000 (S4 Fig and S7 Table). In this smaller-sample scenario, estimation of local genetic correlations and heritability becomes less stable, leading to modest attenuation in the performance of models that rely on these quantities, including PleioSDPR. Even so, PleioSDPR continues to perform comparably to other multivariate PGS models across all genetic-correlation settings, and mtPGS shows stronger performance when sample sizes are small. Overall, these results indicate that PleioSDPR is generally robust to moderate reductions in sample size, although larger sample sizes improve the stability of correlation estimation and enhance predictive performance.”*

3. Page13, line 183. With r2 = 0.9, this simulation scenario likely reflects constant genetic correlations across the genome, which aligns with the underlying assumptions of SDPRX (Page12, line 152). However, in the constant genetic correlations setting (the third column in Figure 1), PleioSDPR_single SDPRX_single should have shown similar performance but PleioSDPR_single outperformed SDPRX_single. Could you explain such difference?

**Answer:** Thank you for raising this important point. Although the scenario with $r_{g}=0.9$ represents a constant genome-wide genetic correlation, there is an additional aspect of the data-generating mechanism that differs from SDPRx’s assumptions. Specifically, SDPRx assumes that the shared genetic effects have the **same variance component** across the two traits. In contrast, PleioSDPR relaxes this constraint and allows the shared component to have **trait-specific variance parameters**, providing greater flexibility in modeling heterogeneous effect-size distributions.

In our simulation design, although the genetic correlation was constant, the two traits were generated with **different heritabilities.** This setting mimics realistic scenarios observed in empirical GWAS, in which, genetically, the heritabilities of two correlated traits differ. Under such conditions, PleioSDPR is better aligned with the true simulation setting. PleioSDPR_single can more accurately capture the underlying effect-size architecture than SDPRx_single, leading to the improved performance observed in the third column of Fig 1.

We have added a clarification in the manuscript to describe this distinction in model assumptions, which is highlighted with a yellow background in the document ‘Revised Manuscript with Track Changes’:  *“We also observed that PleioSDPR, which modeled local genetic correlation and flexible trait-specific variances, outperformed SDPRx, which assumed a constant genetic correlation and equal variances for two traits.”*

4. Page 15, line 224. The number of regions with significant local genetic correlation for trait pairs were calculated from SUPERGNOVA. It seems that the P < 0.05 only means this region have significant local genetic correlation but not emphasis the heterogeneous local genetic correlations. Could you explain how the heterogeneity local genetic correlations are specifically manifested?

**Answer:** Thank you for this insightful comment. You are correct that identifying regions with P < (0.05/number of regions) in SUPERGNOVA indicates statistical significance, but does not by itself convey heterogeneity in local genetic correlations. To demonstrate heterogeneity, we presented the **distribution of the estimated local genetic correlations across all significant regions and across all trait pairs**. As shown in the new supplementary figure 1, the estimated local genetic correlations span a wide range—rather than clustering around a single global value—indicating that different genomic regions contribute differently to the shared genetic architecture.

We have added clarifying text in the manuscript and included the distribution plot to illustrate how heterogeneity manifests across regions. The description is highlighted with a yellow background in the document ‘Revised Manuscript with Track Changes’:

*“We assessed this heterogeneity by examining the distribution of significant local genetic correlations across genomic regions. As shown in S1 Fig, these local estimates span a wide range across regions, demonstrating heterogeneity in regional genetic sharing rather than a single uniform correlation structure.”*

5. Page 26, line 439. Apart from the prediction performance, the comparison of computation efficiency between methods is still important. Please add the corresponding materials to enhance clarity for our readers.

**Answer:** Thank you for highlighting the importance of computational efficiency. We agree that runtime is an important practical consideration. We added a new supplementary table (S8 Table) that reports and compares the runtime of all methods evaluated under a representative simulation scenario. This provides a more quantitative assessment of computational efficiency across approaches. We also expanded the discussion section to explicitly acknowledge the computational cost of PleioSDPR and to note ongoing efforts to improve efficiency, including a potential C++ implementation as shown below, and this description is highlighted with a yellow background in the document ‘Revised Manuscript with Track Changes’:

*“Additionally, the PleioSDPR computational process is rather time-consuming, even when leveraging parallel computation across 22 chromosomes with the allocation of six threads per chromosome. We have quantified this limitation more explicitly in S8 Table, which compares the runtime of PleioSDPR with other competing methods under a representative simulation setting. To enhance efficiency, we are exploring the possibility of rewriting the code using C++ programming.”*

**Minor Comments**

1. Page13, line 179. The expression of genetic correlation r1 appears to conflict with the rk in formula (4) in Page 9, line 119. Please verify these expressions.

**Answer:** Thank you for pointing this out. We have changed the original $r_{1}$ and $r_{2}$ to $r^{share1}$ and $r^{share2}$ in the formular (9) to avoid conflict with the rk in formula (4). The current formula (9) looks like:

$$\binom{\beta_{j1}}{\beta_{j2}}\sim p_{0}\binom{\delta_{0}}{\delta_{0}}$$

+ $p_{1} \binom{N(0,\frac{h_{1}^{2}}{M(p_{1}+p_{3}+p_{4})})}{\delta_{0}}$

+ $p_{2} \binom{\delta_{0}}{N(0,\frac{h_{2}^{2}}{M(p_{2}+p_{3}+p_{4})})}$

+ $p_{3} N\left( \binom{0}{0},\left( {\frac{h_{1}^{2}}{M(p_{1}+p_{3}+p_{4})} \atop r^{shar1}\frac{h_{1}h_{2}}{M\sqrt{(p_{1}+p_{3}+p_{4})(p_{2}+p_{3}+p_{4})}}}{r^{share1}\frac{h_{1}h_{2}}{M\sqrt{(p_{1}+p_{3}+p_{4})(p_{2}+p_{3}+p_{4})}} \atop\frac{h_{2}^{2}}{M(p_{2}+p_{3}+p_{4})}} \right) \right)$

+ $p_{4} N\left( \binom{0}{0},\left( {\frac{h_{1}^{2}}{M(p_{1}+p_{3}+p_{4})} \atop r^{share2}\frac{h_{1}h_{2}}{M\sqrt{(p_{1}+p_{3}+p_{4})(p_{2}+p_{3}+p_{4})}}}{r^{share2}\frac{h_{1}h_{2}}{M\sqrt{(p_{1}+p_{3}+p_{4})(p_{2}+p_{3}+p_{4})}} \atop\frac{h_{2}^{2}}{M(p_{2}+p_{3}+p_{4})}} \right) \right).$

2. Page 21, Line 331. It is hard to observe the difference between methods, especially for those “mul” methods in Figure 1 (also for supplement Figure 1 and 2). Please consider adding the R² values to Figures for better clarity.

**Answer:** Thank you for this helpful suggestion. We agree that annotating the exact $R^{2}$values can improve interpretability. We explored adding the numerical $R^{2}$values directly to the boxplots; however, due to the large number of methods and simulation settings, the figures became visually overcrowded and difficult to read.

To address this concern while maintaining figure clarity, we created Supplementary Tables S4–S7, which report the mean of the exact $R^{2}$values across ten simulations for all methods and simulation scenarios corresponding to Fig 1 and Figs S2 to S4. These tables provide clear numerical comparisons without compromising the readability of the graphical results. We have added references in the figure legends directing readers to the supplementary tables.

3. Page 23, Line 364. “…without the consideration of environmental covariance” means simply rho(e) = 0?

**Answer:** Thank you for the question. Yes, “without the consideration of environmental covariance” means that in our model, we didn’t consider sample overlap. To avoid confusion, we have added the following clarifying sentence to the manuscript, which is highlighted with a yellow background in the document ‘Revised Manuscript with Track Changes’:

“When incorporating environmental covariance, we estimate $s=(N_{s}\rho_{e})/\sqrt{N_{1}N_{2}}$, as described in Supplementary Method S5. When environmental covariance is not considered, we simply set $s=0$, i.e., assume $N_{s}=0$.”

4. Supplement Figures and Tables. The title of Supplement Figure 6 should be corrected as “…have complete sample overlap” but not “…have partial sample overlap”.

**Answer:** Thank you so much for your careful reading. This has been modified in current Fig S8 since we added two more supplementary figures above this figure.

5. Supplement Method. Page 13, Line 1. The formula of rho(t) should be corrected as rho(t) = rho(g) + rho(e).

**Answer:** Thank you again for your careful reading. This has been modified under S5 section in S1 file as:

*”We can estimate* $\frac{N_{s}\rho_{e}}{\sqrt{N_{1}N_{2}}}$ *where* $\rho_{t}=\rho_{g}+\rho_{e}$*is the sum of genetic covariance and environmental covariance.“*

**Respond to Reviewer #2:**

**We sincerely thank you for the constructive and insightful comments. Below, we provide point-by-point responses and describe the corresponding revisions made in the manuscript.**

1. Line 53: does SDPRX model cross-population or cross-trait effects? My understanding was that it is for cross-population.

**Answer:** Thank you very much for pointing this out. Yes, SDPRx is for cross-population and the same as PRScsx. However, after modifying the reference panels to be from the same population and providing the GWAS summary statistics of two traits as input, both methods can also be applied to a cross-trait setting. Conceptually, because these methods are designed to borrow information across correlated effect-size distributions, they can also incorporate information from correlated traits under a cross-trait setting. To clarify this rationale, we have added the following sentences to the manuscript in the introduction part and highlighted them with yellow background in the document ‘Revised Manuscript with Track Changes’:

*“Although many multivariate PGS models were initially developed for cross-population prediction, their underlying modeling frameworks are not restricted to population differences. These methods jointly model multiple GWAS inputs by assuming partially shared effect-size distributions and by applying coupled shrinkage across the input datasets. When the reference panels are applied to the same population, the cross-study information sharing in these models effectively becomes cross-trait sharing. In other words, when the two input GWAS correspond to different traits rather than different populations, the Bayesian effect-size sharing mechanism still allows each trait to borrow statistical strength from the other. This enables PRScsx and SDPRx to function as multivariate PGS approaches in a cross-trait context, leveraging pleiotropy to improve prediction performance.”*

2. Line 55: I assume this should be SDPRX rather than PRS-CSx?

**Answer:** Thank you for your question. The method referenced here is indeed **PRScsx,** not SDPRx. In this part of the manuscript, we intended to discuss the limitations of **JointPRS**, an extension of the PRScs model. Therefore, the appropriate comparison in this context is with PRScsx rather than SDPRx. However, we have removed the discussion of JointPRS from the introduction, since it is not included in our comparison. Instead, we moved the discussion of this method to our discussion section as showing below and highlighted with yellow background in the document ‘Revised Manuscript with Track Changes’.

*“JointPRS [37], which extends PRScsx [10], is another multivariate PGS framework that leverages genetic correlations across multiple populations and can, in principle, incorporate more than two GWAS datasets. Although JointPRS is related in spirit to multivariate PGS modeling, it is primarily designed for settings involving* ***multiple*** *GWAS inputs, whereas the present study focuses specifically on the* ***two-trait*** *cross-trait scenario. Therefore, JointPRS may become a relevant comparison in future extensions of our method that accommodate more than two traits. “*
3. There are several recent methods which leverage multiple cross-trait PGS to improve prediction, e.g. multi-PGS (Albinana et al., 2023 Nat. Comms.) and PRSmix+ (Truong et al., 2024, Cell Genomics). Although these methods work on matrices of PGS rather than starting with summary statistics and also require a validation set, they have the same goal of leveraging correlated traits to improve PGS prediction. How do these methods relate to your current work and is it appropriate to consider them as competing approaches?

**Answer:** Thank you for highlighting multi-PGS (Albiñana et al., Nature Communications, 2023) and PRSmix+ (Truong et al., Cell Genomics, 2024). These methods indeed leverage information across multiple correlated traits to improve prediction performance. However, they differ from our proposed framework in several key respects relevant to the present study. Both multi-PGS and PRSmix+ operate on **matrices of pre-computed PGSs** and require a validation cohort to learn trait-specific weights for prediction. In contrast, PleioSDPR is designed to function **directly on GWAS summary statistics** and does **not require a** validation dataset, making it applicable in settings where only summary-level information is available.

Because these approaches differ fundamentally in their data requirements, modeling assumptions, and intended use cases, we view multi-PGS and PRSmix+ as complementary rather than as direct competitors to our method. Nonetheless, these two approaches are highly relevant to the broader landscape of multi-trait PGS and are therefore appropriate to include in our Discussion section to clarify their relationship to our work and to inform readers about additional multi-trait applications. Accordingly, we have added the following sentences to the Discussion section and highlighted them with a yellow background in the document ‘Revised Manuscript with Track Changes’:

*“Beyond summary-statistic–based models, several recently proposed frameworks—such as multi-PGS [35] and PRSmix+ [36] also aim to leverage correlated traits to improve prediction accuracy. These methods construct large libraries of single-trait PGS and require a validation cohort to learn optimal trait-specific weights. While highly effective in data-rich biobank settings, these approaches do not operate directly on GWAS summary statistics. PleioSDPR, in contrast, is designed for scenarios where only summary-level information is available and focuses specifically on modeling two traits through shared and region-specific genetic architecture without using a validation dataset. Thus, multi-PGS and PRSmix+ are complementary rather than directly comparable to PleioSDPR.”*

4) Line 101: a closing bracket appears to be missing. Also, what does j refer to here? Later it indexes SNPs, but it doesn’t seem to do that here.

**Answer:** Thank you for your careful reading. Since this is a piecewise function, there may not be a need for a closing bracket. In our revised manuscript, we have changed j into $i_{1} and i_{2}$ and explained their meaning in both the main manuscript (highlighted with a yellow background in the document ‘Revised Manuscript with Track Changes’) and the Supplementary Method as follows:

*“…., where* $i_{1}$ *and* $i_{2}$ *are the individual indices for the first and second GWAS, respectively, assuming that the first* $N_{s}$*samples overlap:*

$$cov\left( \epsilon_{1,i_{1}},\epsilon_{2,i_{2}} \right)=\left\{ \begin{aligned} \rho_{e} , \\ 0 , \end{aligned} \right.\begin{matrix} 1\leq i_{1}=i_{2}\leq N_{s} \\ otherwise \end{matrix}$$

(2)

*“*

5) Line 113: you choose to set a to 0.1. Is there a strong justification for choosing this value? How would other values affect the results and how should one choose this value?

**Answer:** Thank you for pointing this out. First, we apologize for the confusion—the value we use in all analyses is $a=0.1$, and the notation “$Na=0.1$” in the earlier draft was incorrect. This has been fixed in the revised manuscript, highlighted with a yellow background in the document ‘Revised Manuscript with Track Changes’.

# Our choice of $a=0.1$follows the original SDPR formulations **(A fast and robust Bayesian nonparametric method for prediction of complex traits using summary statistics),** where the likelihood includes the regularized covariance term

$(R+NaI)/N$,

with $Na=0.1$ a suggested default to reduce sensitivity to LD mismatch between the GWAS summary statistics and the reference panel. Since we simplified the related likelihood formulation in PleioSDPR as:

${\hat{\beta}_{1}| \atop\hat{\beta}_{2}|}{\beta_{1} \atop\beta_{2}}\sim N\left( \binom{D\beta_{1}}{D\beta_{2}},\left( \begin{matrix} (D+aI)/N_{1} & N_{s}\rho_{e}(D+aI)/N_{1}N_{2} \\ N_{s}\rho_{e}(D+aI)/N_{1}N_{2} & \left( D+aI \right)/N_{2} \end{matrix} \right) \right)$,

$Na$ in SDPR is then equal to a in our formulation. Conceptually, the parameter $a$ applies a mild regularization that shrinks the off-diagonal elements of the covariance matrix, helping stabilize effect-size estimation for SNPs in high LD, especially when the LD reference may not perfectly match the GWAS population.

In our setting, we adopt the same default value $a=0.1$ to maintain consistency with SDPR—the parent model on which PleioSDPR is built—and to avoid introducing an additional tuning parameter. We did not observe instability or over-shrinkage in either our simulation settings or real-data analyses under this choice. Throughout our simulation and real-data analyses, we noted that, in practice, larger values of $a$ would impose stronger shrinkage on correlated SNPs, whereas smaller values closer to zero would provide minimal adjustment.

6) How is PRS-CSx used in this work? It appears that only EUR samples were used for the GWAS, wheras PRS-CSx is traditionally used with sumstats from multiple ancestries. Were the two traits treated as if they were from different ancestries, while using the same reference panel?

**Answer:** Thank you for your questions. Even though PRScsx is for cross-population, after modifying the reference panels to be from the same population and providing GWAS summary statistics for two traits from the same population as the reference panel, it can also be applied to a cross-trait setting. Because PRScsx was designed to borrow information across correlated effect-size distributions, it can also incorporate information from correlated traits under a cross-trait setting.

7) Similarly, JointPRS is mentioned in the introduction, but not used as a comparison method. If multiple ancestry sumstats are indeed being used, this would be odd to leave out after mentioning it in the intro.

**Answer:** Thank you for pointing this out. JointPRS was designed for scenarios that involve **more than two** GWAS datasets, whereas our manuscript focuses specifically on the **two-trait cross-trait** prediction setting. For this reason, we did not include JointPRS in our comparison at this stage. You are also correct that its mention in the Introduction would be odd, so we now discuss JointPRS and its potential future application in the Discussion section which is highlighted with yellow background in the document ‘Revised Manuscript with Track Changes’.

*“JointPRS [37], which extends PRScsx [10], is another multivariate PGS framework that leverages genetic correlations across multiple populations and can, in principle, incorporate more than two GWAS datasets. Although JointPRS is related in spirit to multivariate PGS modeling, it is primarily designed for settings involving* ***multiple*** *GWAS inputs, whereas the present study focuses specifically on the* ***two-trait*** *cross-trait scenario. Therefore, JointPRS may become a relevant comparison in future extensions of our method that accommodate more than two traits. “*

8) It seems unnecessary to use 3 differently sized validation sets if the R2 values are ultimately averaged. Is there a reason for this?

**Answer:** Thank you for asking. We used three validation datasets of different sizes to ensure that our parameter tuning was not overly dependent on any single validation sample and to evaluate the robustness of each method to variation in validation-set size. In practice, the choice of validation sample size can influence hyperparameter selection—particularly for methods such as PRScs, PRScsx, LDpred2, and mtPGS, which rely on validation data for tuning. By using validation datasets of varying sizes, we aimed to mitigate potential instability in hyperparameter selection and obtain a more reliable, less sample-size–specific estimate of predictive performance. Averaging the resulting $R^{2}$values provide a stable summary measure that reflects performance across a range of plausible validation conditions, rather than depending on a single arbitrary split.

We have clarified this rationale in the revised manuscript as follows and highlighted it with a yellow background in the document ‘Revised Manuscript with Track Changes’.

*“To reduce potential dependence on any single validation dataset and to assess the robustness of hyperparameter tuning, we randomly partitioned these individuals into three validation datasets of different sizes. Methods such as LDpred2, PRScs, PRScsx, and mtPGS rely on a validation dataset for tuning, and the optimal hyperparameters can vary with the size of the validation sample. Using multiple validation sets of varying sizes helps mitigate instability arising from a specific split and provides a more reliable assessment of each method’s tuning performance.”*

9) Line 340: The calculation of R2 deserves a bit more explanation. e.g. Do you control for PCs? Is this the R2 attributable to the PRS alone or to the full model with covariates?

**Answer:** Thank you for raising this point. In our analysis, the reported $R^{2}$ values correspond to the variance explained by the PRS **alone**, without adjusting for any additional covariates. We have clarified this in the revised manuscript as follows and highlighted it with a yellow background in the document ‘Revised Manuscript with Track Changes’:

*“To evaluate predictive performance, we fit a simple linear regression model of the form* $\text{phenotype}\sim\text{P}\text{G}\text{S}$ *in the testing dataset and reported the resulting coefficient of determination (*$R^{2}$*), which reflects the variance explained by the PGS alone. This procedure yielded three* $R^{2}$*values—one for each validation-derived model—and their mean served as our final evaluation metric.”*

10) For real data figures, confidence intervals should be included around point estimates for R2.

**Answer:** Thank you for this helpful suggestion. We have now added 95% confidence intervals to all real-data figures. The revised figures now display error bars reflecting these intervals. We have also included the description of these error bars under the figures.

Minor points:
1) Line 26: Usually, the term bipolar “disorder” is preferred rather than “disease”.

**Answer:** Thank you for pointing this out. We have changed all “bipolar disease” to “bipolar disorder” in the updated manuscripts.

2) Line 316: Should this read “complete” instead of “no” sample overlap?

**Answer:** Thank you for your careful reading. We have corrected this typo. In the current “Manuscript” file, it shows “*However, this advantage is less obvious in cases with complete sample overlap between two traits (S3 Fig and S6 Table).”* at line 393

**Respond to Reviewer #3**

**We sincerely thank you for the constructive and insightful comments. Below, we provide point-by-point responses and describe the corresponding revisions made in the manuscript.**

• Introduction: SDPRX is introduced with several PGS methods focusing on a single trait. It fits better with PleioPred and mtPGS which construct PGS considering correlated traits.

**Answer:** Thank you for your suggestion. We have revised the Introduction to clarify why SDPRx and PRScsx—although initially developed for cross-population prediction—can also be applied in settings with correlated traits, as both methods leverage shared genetic architecture across multiple GWASs. Since PRScsx is an extension of PRS-cs, we have a brief introduction on this. We have also expanded the discussion of PleioPred and mtPGS to more clearly introduce these methods as multivariate PGS approaches designed to incorporate information from genetically correlated traits. We have added the following sentences to the manuscript in the introduction part and highlighted with a yellow background in the document ‘Revised Manuscript with Track Changes’:

*“Although many multivariate PGS models were initially developed for cross-population prediction, their underlying modeling frameworks are not restricted to population differences. These methods jointly model multiple GWAS inputs by assuming partially shared effect-size distributions and by applying coupled shrinkage across the input datasets. When the reference panels are applied to the same population, the cross-study information sharing in these models effectively becomes cross-trait sharing. In other words, when the two input GWAS correspond to different traits rather than different populations, the Bayesian effect-size sharing mechanism still allows each trait to borrow statistical strength from the other. This enables PRScsx and SDPRx to function as multivariate PGS approaches in a cross-trait context, leveraging pleiotropy to improve prediction performance.”*

*“PleioPred leverages pleiotropy by assuming that the true SNP effect sizes follow a mixture distribution that accommodates four possibilities: a SNP may be causal for both traits, causal for only one trait, or non-causal for either trait. However, PleioPred assumes a simplified form of shared genetic architecture between traits and does not incorporate genetic correlations. In addition, it lacks an auto version for scenarios where a validation dataset is unavailable. mtPGS models the joint distribution of effect sizes across two traits under the assumption of a constant, genome-wide genetic correlation. However, it does not account for local genetic correlations and does not explicitly model SNPs with null effects on both traits and trait-specific null effects. Therefore, there is a need for new methods that more fully leverage information from correlated traits by incorporating local genetic heritability and local genetic correlations, while maintaining strong performance in settings without a validation dataset.”*

• Introduction: line 63 “follow a four-component normal distribution” it is helpful to briefly describe these four components to understand the method’s unique feature.

**Answer:** Thank you for your suggestion. We have added more descriptions to introduce the features of PleioPred. The modified version is shown below and is highlighted with a yellow background in the document ‘Revised Manuscript with Track Changes’:

*“PleioPred leverages pleiotropy by assuming that the true SNP effect sizes follow a mixture distribution that accommodates four possibilities: a SNP may be causal for both traits, causal for only one trait, or non-causal for either trait.”*

• Methods: line 115-116 “the minimal sample sizes of the individuals from the same cohort of two traits as the input of overlap sample size” the authors need to clarify what “minimal sample sizes” means, probably with some notations for two traits (e.g., Ny1 and Ny2 indicate the sample size of trait y1 and y2, respectively).

**Answer:** Thank you for your suggestion. We have revised the manuscript to include a more detailed explanation—using explicit notation—of how the overlapping sample size is determined, as described below, and the description is highlighted with a yellow background in the document ‘Revised Manuscript with Track Changes’:

*“If the exact overlap sample size cannot be determined, we recommend using* $min(N_{1}^{A},N_{2}^{A})$*— the minimum of the sample sizes contributed by the same cohort A to the GWASs of traits* $Y_{1}$ *and* $Y_{2}$*—as an approximation of the overlap sample size.”*

• Methods: for equation (4), it would be helpful to justify why k is capped at 1,000. Recent large GWAS have reported a large number of significant loci > 1,000. What would be the impact if k >1,000? If it is an arbitrary choice of computational parameters, maybe ranking these significant genetic associations could minimize the overall impact of the performance.

**Answer:** Thank you for your insightful comment. In PleioSDPR, we follow the design of SDPRx, which uses a truncated stick-breaking process to approximate a Bayesian nonparametric prior. As described in SDPRx and in our Methods section, truncation is required to make the mixture model computationally feasible, and the choice of 1,000 components serves as an upper bound rather than a biologically meaningful limit.

Although recent large-scale GWASs have identified more than 1,000 genome-wide significant loci, the stick-breaking representation does not require each component to correspond to a single locus. Instead, each component captures groups of SNPs that share similar genetic variances and covariances. In practice, only a subset of the 1,000 components receives non-negligible posterior weight. This was also observed in SDPRx, where the number of “active” components—that is, components to which SNPs were assigned—was far fewer than 1,000. Thus, increasing the truncation level beyond 1,000 has minimal impact on performance while substantially increasing computational cost.

To clarify this point, we have added the following explanation in the Methods section, which is highlighted with a yellow background in the document ‘Revised Manuscript with Track Changes’:

*“Following SDPRx, PleioSDPR also uses a truncated stick-breaking process [12,18] to represent the probability of assignment for the second (trait1-specific), third (trait2-specific), and fourth terms (shared with correlation), and applies truncation by setting the maximum number of mixture components to 1,000. This value serves as a computational upper bound, and in practice, only a small subset of components receives appreciable posterior mass, and increasing the truncation level of the components provides no meaningful performance gain while substantially increasing computational cost.”*

• Methods: line 140-141 “We partitioned the LD matrix into approximately independent LD blocks to reduce the computational burden”. How many LD blocks were calculated? The number is dependent on the LD threshold and ancestry populations.

Answer:

**Answer:** Thank you for your question. Our LD-block construction partitions each chromosome into approximately independent segments based on an LD threshold of $r^{2}>0.1$. Since our simulation and real-data analysis focus on the European population, we used the EUR 1KG as our reference panel. So, the LD threshold, together with the LD structure of the European reference population, determines the resulting number of blocks in this manuscript.

Across chromosomes 1–22, our pipeline generated 1,617 blocks in total. These block counts are now reported in the Methods section for clarity, which is highlighted with a yellow background in the document ‘Revised Manuscript with Track Changes’:

*“We partitioned the LD matrix into approximately independent LD blocks to reduce the computational burden (e.g., 1,617 blocks with an LD threshold of* $r^{2}>0.1$ *and the 1000 Genome Phase 3 EUR data as reference panel).”*

• PGS method comparison: PleioPred and mtPGS were two very relevant methods which consider correlated traits, similar to PleioSDPR. However, the authors chose not to include them for comparison. The authors need to include them for comparison or clearly justify why they were not considered for comparison.

**Answer:** Thank you for raising this important point. We have now included mtPGS in both our simulation studies and real data analyses to provide a more comprehensive comparison with existing multivariate PGS approaches.

Regarding PleioPred, we appreciate its relevance and introduce it in our Introduction section; however, we did not include it in our comparison for the following reason. PleioPred does not provide an auto-tuning or validation-free version. A central focus of our manuscript is evaluating prediction accuracy in the absence of a validation dataset, as this is a common scenario in practical applications and one in which existing methods often differ most substantially. Because PleioPred requires a validation set for tuning, it cannot be fairly compared under this setting. We also added more explanation on the limitations of PleioPred in the Introduction Section as follows, which is highlighted with a yellow background in the document ‘Revised Manuscript with Track Changes’:

*“However, PleioPred assumes a simplified form of shared genetic architecture between traits and does not incorporate genetic correlations. In addition, it lacks an auto version that can be used in scenarios where a validation dataset is not available.”*

• Heritability estimate: seems the authors used LDSC to calculation SNP heritability from GWAS. LDSC is a convenience tool but know to underestimate the trait heritability. It may also cause the discrepancy of heritability of the same traits from different GWAS (e.g., WC from GIANT vs. UKB.)

**Answer:** Thank you for this valuable comment. We agree that SNP heritability estimates derived from LDSC are known to be conservative and may underestimate the true heritability. LDSC is also sensitive to GWAS characteristics such as sample size, LD structure, and ascertainment differences, which can lead to discrepancies in estimated heritability across GWAS of the same trait.

In our manuscript, heritability estimated by LDSC was primarily used for **descriptive summarization** of trait characteristics rather than as input to model fitting or parameter tuning. The heritability values were not used to calibrate any part of PleioSDPR; therefore, potential downward bias in LDSC estimates does not affect the comparative evaluation of PGS methods. We chose LDSC because it is widely used, computationally efficient for large GWAS, and provides consistent estimates across traits under a unified framework.

We have added clarification in the manuscript as follows to note that LDSC tends to underestimate heritability, and the description is highlighted with a yellow background in the document ‘Revised Manuscript with Track Changes’:

*“We report SNP heritability estimated from LDSC [26] for descriptive purposes. As LDSC is known to produce conservative heritability estimates and may yield different values across GWAS due to differences in sample size, LD structure, or phenotype measurement, these estimates were not used to tune any model parameters and do not affect the comparative evaluation of PGS methods.”*
